# Mental Rotation: Effects of Gender, Training and Sleep Consolidation

**DOI:** 10.1371/journal.pone.0060296

**Published:** 2013-03-27

**Authors:** Ursula Debarnot, Pascale Piolino, Jean-Claude Baron, Aymeric Guillot

**Affiliations:** 1 Centre de Psychiatrie et Neurosciences (Inserm UMR S894), Université Paris Descartes, Paris, France; 2 Laboratoire Mémoire et Cognition, Institut de Psychologie, Boulogne Billancourt, France; 3 Centre de Recherche et d’Innovation sur le sport, EA 647, Université Claude Bernard Lyon 1, Université de Lyon, France; 4 Laboratoire de Neuropsychologie et du Vieillissement, Institut de Psychologie, Boulogne-Billancourt, France; 5 Institut Universitaire de France, Paris, France; University of São Paulo, Brazil

## Abstract

A wide range of experimental studies have provided evidence that a night of sleep contributes to memory consolidation. Mental rotation (MR) skill is characterized by fundamental aspect of both cognitive and motor abilities which can be improved within practice sessions, but little is known about the effect of consolidation after MR practice. In the present study, we investigated the effect of MR training and the following corresponding day- and sleep-related time consolidations in taking into account the well-established gender difference in MR. Forty participants (20 women) practiced a computerized version of the Vandenberg and Kuse MR task. Performance was evaluated before MR training, as well as prior to, and after a night of sleep or a similar daytime interval. Data showed that while men outperformed women during the pre-training test, brief MR practice was sufficient for women to achieve equivalent performance. Only participants subjected to a night of sleep were found to enhance MR performance during the retest, independently of gender. These results provide first evidence that a night of sleep facilitates MR performance compared with spending a similar daytime interval, regardless gender of the participants. Since MR is known to involve motor processes, the present data might contribute to schedule relevant mental practice interventions for fruitful applications in rehabilitation and motor learning processes.

## Introduction

Mental rotation (MR) is a mental operation during which a mental image is rotated around some axis in three-dimensional space [1]. In typical Shepard–Metzler MR paradigm, participants are asked to compare pairs of objects rotated in 3D space relative to one another, and to determine whether they are similar or mirror images. This MR task usually generates an extremely robust set of behavioral findings, with the time taken to respond to each stimulus pair increasing with the angle of rotation between them [2], [3]. Likewise, MR is a visuospatial task which gives rise to robust gender-based differences in performance, with men usually outperforming women [4], [5]. In particular, important gender differences were observed with the Vandenberg and Kuse MR test (VK) [6], which consists of multiple 3D-MR cubes within a single problem. Although gender differences in performance on the classic Shepard–Metzler task diminished after 3D-MR training [7], it still remains to see whether such gender-training effect in MR performance occurs with the VK test. Interestingly, Jordan et al. [8] suggested that environmental factors may interact with possible different gender-related strategies. Accordingly, men might use a visuo-spatial holistic strategy in which the object is pictured in the mind and then rotated, while women would rather prefer verbal or analytical strategies resulting in a more piecemeal MR process [9], [10]. Consistently, functional neuroimaging studies investigating gender differences in MR tasks revealed that women engage areas involved in spatial working memory (WM), hence suggesting more effortful “top–down” processing, while men primarily yield a visuomotor network reflecting more efficient “bottom–up” processing [11], [12], [13]. Besides, there is now ample evidence that motor areas are activated during MR of hands or objects [14]. For example, using transcranial magnetic stimulation, Eisenegger and collaborators [15] demonstrated that MR of standard Shepard and Metzler figures activate the primary motor cortex (including the corresponding corticospinal pathway) as indicated by an increase in motor evoked potentials.

It is now well-established that post-learning sleep is beneficial for human memory performance through a so-called “system consolidation process”. During such off-line process, a labile memory trace is known to become more robust to decay, or even improve recollection [16]. Sleep-dependent skill learning has been demonstrated across a wide variety of skill domains, including perceptual [17], [18], pitch memory [19] and sequential motor practice [20], [21], [22], and even following mental practice [23], [24], [25]. A substantial body of evidence further demonstrated that the pattern of brain activity elicited while practicing a memory task actually reemerges or is replayed during sleep [26], [27], [28]. Such replay of memory in the sleeping brain allows not only the maintaining or strengthening of item memories, but also facilitates the off-line assimilation and generalization of individual memories in a manner that optimizes their potential usefulness in facing the future [29], [30]. Accordingly, Kuriyama et al. [31] demonstrated a sleep-dependent improvement of WM performance and further concluded that post-training sleep could be a potent facilitating factor in WM capacity, leading to the advancement of individual general fluid intelligence [32]. These data emphasize the importance of sleep in learning many real-life motor skill routines. Taken together, there is substantial evidence that sleep serves a meta-level role in memory processing that moves far beyond the consolidation and strengthening of individual memories, and further aims to intelligently assimilate and generalize these details offline [33].

Although several studies reported that men perform better than women in MR task using different strategies [9], [11], which are reflected at a neural level [7], [10], [13], gender differences have been less well investigated in the field of sleep and memory consolidation research. To date, only two studies explored the gender effect in nap memory consolidation process, though using declarative-learning paradigms. Genzel et al. [34] first explored the effect of nap on memory consolidation both on procedural and declarative tasks previously learnt by men and women either during early-follicular phase or during mid-luteal phase of the menstrual cycle. They found that men performed significantly better on the two memory tasks after a nap, while women improved performance only in the mid-luteal phase, suggesting that sleep-related memory consolidation has a higher complexity and more influencing factors than previously assumed. Nevertheless, it has been reported that procedural consolidation, expressed as an off-line motor skill improvement, can be blocked by declarative learning over wake, but not over a night of sleep, and *vice versa* [35], [36]. Unfortunately, the interfering effect between the two memory systems during the day, and their independence during sleep, were not considered in the study of Genzel et al. [34]. In a second study, Wang et al. [37] investigated the effects of daytime sleep on episodic declarative memory, and recollection and familiarity in women and men groups. They reported that a nap may contribute to the retention of the contextual aspect of episodic memory rather than the item information itself in women, while nap increased familiarity in men. Such gender difference may be linked with different memory traces resulting from different encoding strategies, as well as different electrophysiological changes during daytime sleep. Finally, Wang et al. [37] concluded that women are more likely to be affected by the influences of daytime sleep than men. Together, these findings provide insights in the characterization of the consolidation process with respect to gender, even though knowledge on this issue is still limited.

Overall, despite accumulated evidence that sleep facilitates the memory consolidation process, there is yet no experimental data about its possible effect following MR practice, on the one hand, nor with respect to gender, on the other. Hence, the main objective of the present study was twofold: *i)* evaluating the effect of a short MR training program, using the 3D-VK test, in both women and men performance, and *ii)* investigating whether delayed performance gains after a night of sleep might occur following MR practice in both groups, compared to daytime consolidation. Many authors promote the use of MR paradigms in virtual reality and neuro-rehabilitation programs [38], [39], [40], [41], with extension to the brain injured of the use of mental images which is critical in cognitive tasks involving memory, reasoning and problem solving in everyday life [42], [43]. Therefore and given that motor processes are involved even when abstract objects are mentally rotated [44], exploring in greater details the effect of both training and off-line consolidation processes of MR might shed light on how optimally scheduling and using MR efficiently from the everyday life situation up to rehabilitation programs. We predicted that both men and women would demonstrate a significant improvement in performance following a MR training session, whereas only participants subjected to a night of sleep were expected to show delayed performance gains.

## Materials and Methods

### Participants

Forty healthy volunteers (20 women, age range: 18–40 years, mean ± SD: 26.5±6.9; 20 men, age range: 21–40 years, mean: 28.3±5.6) participated in this experiment. Both men and women were respectively assigned into two different groups, with one group being subjected to a night of sleep and the other group to a similar daytime delay. Participants were therefore assigned into four different groups (n = 10), i.e. Women-Day (WDay), Women-Night (WNight), Men-Day (MDay) and Men-Night (MNight) groups. All participants were right-handed as assessed by the Edinburgh Handedness Inventory [45], and had normal or corrected to normal visual acuity. They reported sleeping regularly between 7 and 9 h per night. Extreme evening- and morning-type individuals, as well as regular nappers and smokers, were excluded. None of them reported a prior history of drug or alcohol abuse, neurological, psychiatric, or sleep disorders. Additionally, they were asked to be drug, alcohol, and caffeine free for 24 h prior, and during the experiment. To avoid participants with previous experience on MR tasks, we excluded those with a profession requiring high visuo-spatial abilities (e.g. architect, graphic designer). None of the participants had any prior knowledge of the experimental MR design and task. This study was approved by the Ethics Committee of the University Paris Descartes, and all participants signed a written informed consent form. The experimental procedure was explained and participants received instructions about the task, but no information was provided about the objectives of the study or the variables of interest. All participants received course credits required as part of their studies.

Before the experiment, participants filled in the Pittsburg Sleep Quality Index [46] to assess sleep quality and quantity. This test was administered to exclude individuals experiencing obvious disturbances during their sleep-wake cycles and to ascertain predisposition to benefit from the natural effects of sleep. They also completed the Visual Gnosia Evaluation Protocol [47] and the Visual Object and Space Perception [48], which respectively provided an evaluation of the visual discrimination and a basic measure of visuospatial abilities. All participants scored normally on these tests (cutoff<8). Finally, they were asked to fill in the Stanford Sleepiness Score (SSS) [49], a questionnaire providing a subjective measure of alertness. The SSS is a 7-point scale, with 1 being the most alert state. This questionnaire was presented twice during the experiment, i.e., before the MR training session and right before the re-test session (see below).

### Design and apparatus

#### Mental rotation task

**Figure 1:**
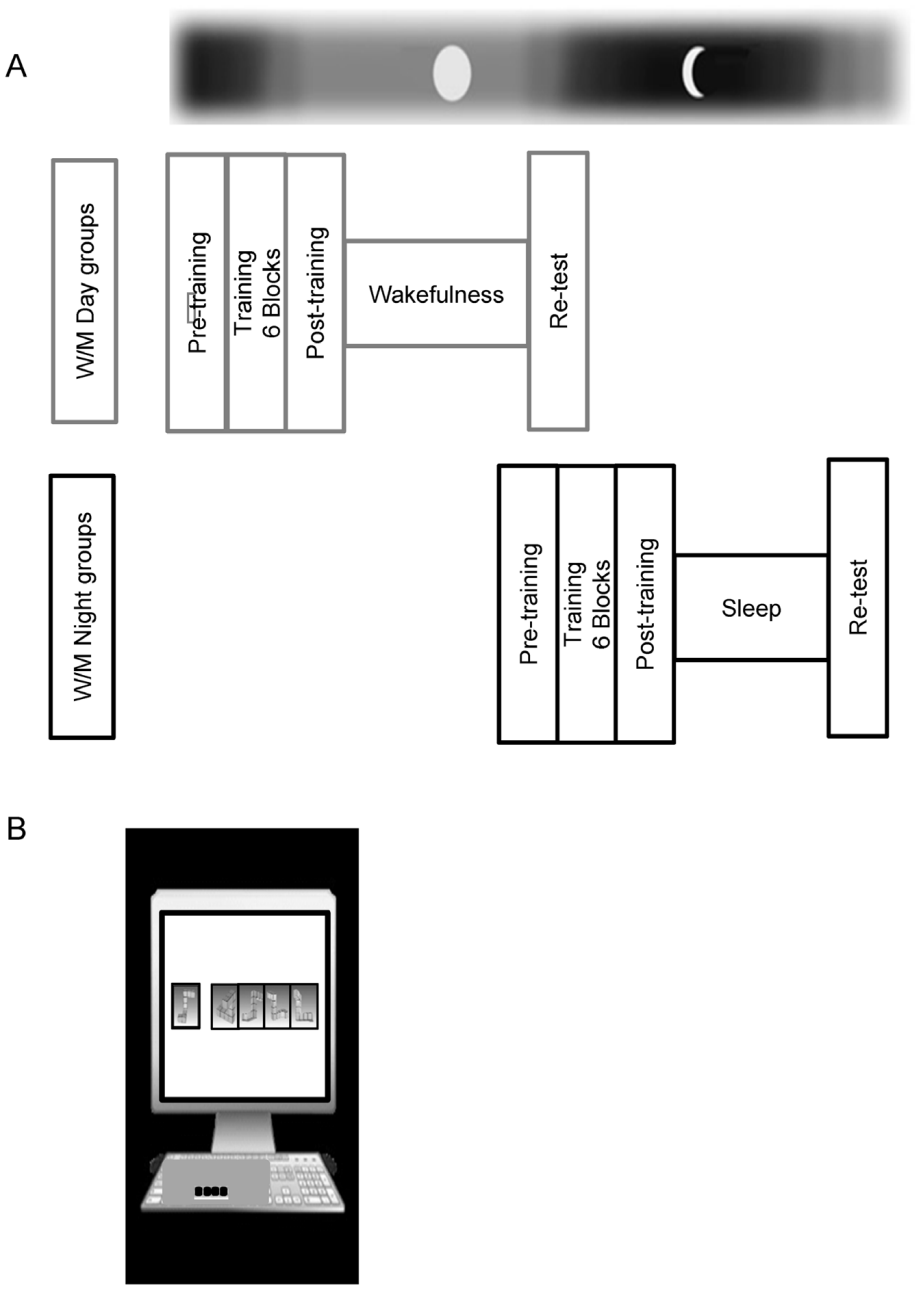
Protocol of the experiment. (A) Schematic view of the experimental protocol. All participants performed a MR practice session including a pre-training (baseline), a MR training phase, and a post-training session, to assess the effect of MR training. Then, half of the participants were re-tested following a 12 h interval including either a night of sleep (WNight/MNight) or a similar daytime period (WDay/MDay), to test the offline consolidation [Re-test/Post-training]. (B) Illustrations of the setup and design of the MR Task.

Participants seated on a chair at a distance of 50 cm in front of a 17-inch computer screen. They were in a quiet room, without any distracting stimuli, in order to help them focusing on the MR task. A computerized 3D version of the MR task elaborated by Vandenberg and Kuse (VK) [6] was used to measure MR learning. The VK MR task was chosen as large and reliable gender differences were consistently reported [50]. This task consists of a series of test items with a target stimulus derived from the Shepard and Metzler figures [1], and four sample stimuli that are arranged to the right of the target figure. Two of them are identical but rotated copies of the target stimulus. Participants were instructed to identify the two sample stimuli that matched the target, and press the corresponding key on a keyboard, as fast and as accurately as possible. The target and reference stimuli appeared in a size of 7.5 cm in diameter on a black screen. Four keys (C, V, B, N of an AZERTY keyboard) were dedicated to the four sample stimuli. The VK MR task is made of 24 items, and each item has two and only two correct sample stimuli that match with the target stimulus. We composed a pre-training block with 12 randomized items, which were the same used for the post-training and re-test; the items appeared in a random order. The six training blocks were composed with the other different items. Each trial started with a black screen for 3 s, where all items (one target and four sample figures) were presented simultaneously. Images remained 30 s until the two responses were carried out. All block sessions were organized according to a trial design alternating a 30 s period for items presentation followed with 3 s of rest indicated by a black screen. Each 12 items block was separated by a rest period of 2 min. Response latencies and accuracy were recorded with the E-Prime experimental software (Version 1.2, Pittsburgh, PA, USA) and constituted dependent variables of interest.

One point was given if both correct images were picked, as suggested by Peters [51].

#### Experimental procedure

Both WDay and MDay groups (n = 10) were subjected to MR training at 8:00 am and were retested on the same day at 8:00 pm, while WNight and MNight groups were first trained in the evening at 8:00 pm and then retested the next morning at 8:00 am (Fig. 1). The experiment was divided into four phases:

#### Familiarization session

During this session, instructions were given to participants who had to find the two correct answers that matched the target item, as fast and accurately as possible. They were asked to perform successively 3 corrected items to familiarize themselves with the task and the stimuli.

#### MR session 1

It consisted of eight practice blocks including 12 items. Practice blocks were separated by a 2 min rest period during which participants were allowed to relax their eyes and stretch out hand and fingers. The 12 items composing the first (pre-training) and the last (post-training) practice blocks were the same, but they were presented in a randomized order. The amount of learning was evaluated by comparing performances between the first (pre-training) and last (post-training) practice blocks.

#### Delay

The MR training session was followed either by an 8 hour (±1 h) night of sleep (the re-test session was scheduled 2 hours after waking up) or a similar daytime period.

#### Re-test session 2

After the consolidation intervals (delay), participants performed a 12 items block in order to evaluate the respective effects of daytime and sleep on MR performance. The items were the same than those used in the pre and post-training blocks, but there were presented in a different randomized order. Performance was compared with that of the post-training session to evaluate the MR off-line consolidation. At the end of this session, participants were given questions related to introspection about processes and strategies used while performing MR. All questions are listed in the Appendix S1.

### Data Analysis

We first checked the Gaussian distribution of the data as well as the homoscedasticity. The sphericity assumption was further tested using the Mauchly’s sphericity test. Data showed that the sphericity was not violated, and thus that parametric statistical tests could be safely used. For each test session (pre-training, post-training and re-test), we analyzed two dependent variables, namely response accuracy (total number of correct answers) and response latencies (mean time lasting from the apparition of the MR item to the second key pressed). To compare the level of performance between women and men at the beginning (first block) and at the end of training (eighth block), we performed two one-way Analyses of Variance (ANOVAs) with session (pre- and post-training) as within-subject factor and gender (Men vs. Women) as between-subjects factor (Statistica, StatSoft Inc., USA). A repeated measures ANOVA (ANOVArm) with gender as between-subject factor and session (pre-training, post-training) as within-subject factor was then used to investigate the effect of training. Finally, to investigate the effect of the consolidation process, we performed an ANOVA_RM_ with gender and consolidation time (Night vs. Day) as between-subjects factors, and session (post-training and re-training) as within-subjects factor. Results are presented as mean (±standard deviation - SD), and threshold for significance was set at p<0.05 and sized effect was indicated. When appropriate, corrected Bonferroni post-hoc comparisons were performed.

## Results

### Questionnaires

The average sleep score, as measured by the PSQI, was 2.85 (±0.97), thus attesting for the ‘good quality’ of sleep in all participants. There was no difference in SSS ratings between sessions or among groups. On the 7-points scale (1 = being most alert), mean values for the MNight group were 2.10 (±0.57) during the first session and 2.10 (±0.19) during the second session. Corresponding scores in the MDay group were 2.20 (±0.63) and 2.40 (±0.21), 2.30 (±1.06) and 1.90 (±0.35) in the WNight group, 2.60 (±0.70) and 2.30 (±0.23) in the WDay group.

### Behavioral data

#### Practice-dependent learning

**Figure 2:**
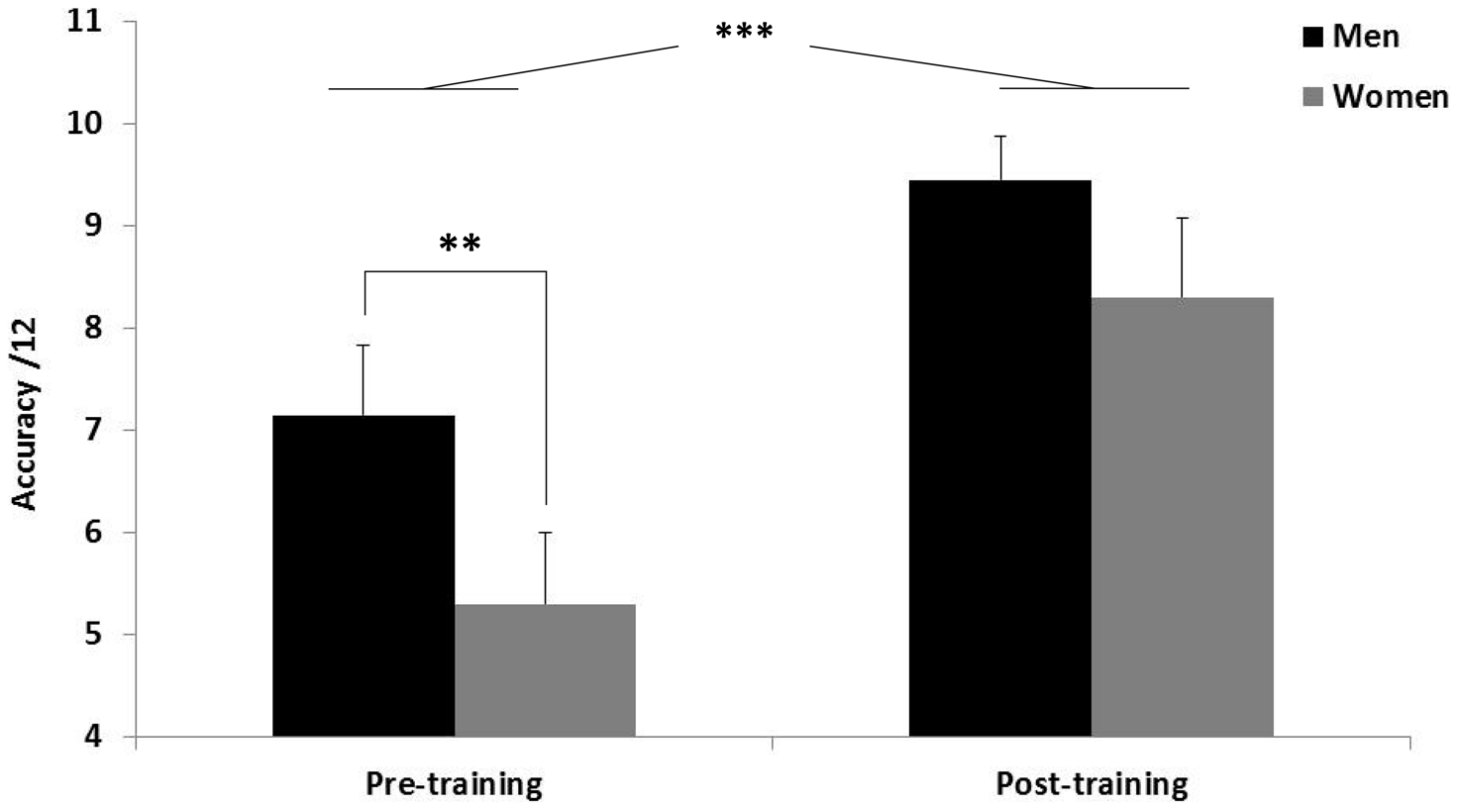
Effect of 3D-KV MR training with respect to gender. The total number of correct answers before and after the MR training has been computed separately for the men (MNight/MDay) and women (WNight/WDay) groups. Men significantly outperformed women at the pre-training test, while training allowed women to reach an equivalent level of performance than men (post-training test). Error bars indicate one SD.

First, we aimed to determine whether women and men were comparable in terms of performance during the pre-training. In this session, women and men respectively performed 5.30±0.49 and 7.15±0.51 correct sequences (one point for two correct answers per item, n = 20). Corresponding mean (±SD) sequence durations were 15.44±1.09 s and 13.84±0.88 s, respectively (Table S1). A one-way ANOVA on the number of correct answers yielded a significant effect of gender in favor of men (F_1,38_ = 7.11, P<0.01, *η_p_* ^2^ = 0.15, Fig. 2), while there was no difference when comparing mean response latencies (F_1,38_ = 1.37, P = 0.24, *η_p_* ^2^ = 0.03).

The number of correct items during post-training was 9.45 (±0.44) in the men group and 8.30 (±0.78) in the women group. An ANOVA_RM_ with gender as between-subject factor and session (pre-training, post-training) as within-subject factor on the number of correct items revealed a main effect of gender (F_1,38_ = 7.77, P<0.01, *η_p_* ^2^ = 0.16) as well as a main session effect (F_1,38_ *= *48.47, P<0.0001, *η_p_* ^2^ = 0.56), but no group x session interaction (F_1,38_ *= *0.84, P = 0.36, *η_p_* ^2^ = 0.02). These data show an improvement of performance from the pre-training to the post-training session, independently of gender, but the gender difference remained stable over practice. Finally, the one way ANOVA on post-training scores did not yield any significant gender effect (F_1,38_ = 3.41, P = 0.07, *η_p_* ^2^ = 0.08), indicating that while men and women’s MR performances were different at the pre-test, training allowed women to reach a level of performance equivalent to that of men at the post-training session.

A slightly different pattern of results was observed when looking at response latencies. The ANOVArm revealed no significant session effect (F_1,38_ = 0.92, P = 0.34, *η_p_* ^2^ = 0.02), no gender effect (F_1,38_ = 2.83, P = 0.10, *η_p_* ^2^ = 0.06), as well as no group x session interaction (F_1,38_ = 0.07, P = 0.79, *η_p_* ^2^ = 0.001). The additional one way ANOVA on post-training latencies did not reveal any significant gender effect (F_1,38_ = 3.40, P = 0.07, *η_p_* ^2^ = 0.08).

#### Sleep-dependent learning

**Figure 3:**
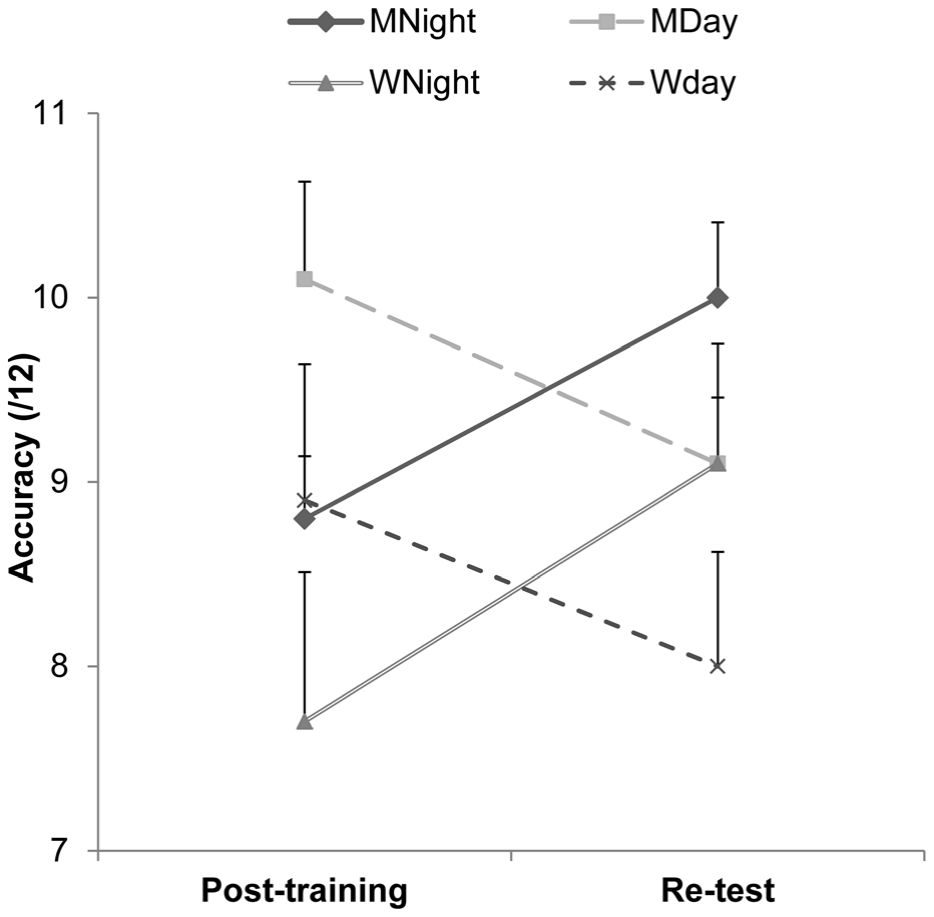
Effect of consolidation on the total number of correct answers. The total number of correct answers performed during the post-training and the retest sessions have been computed for the four different groups of participants. Night groups significantly increased their performance in the re-test session, while a decrease in performance was found in Day groups, thus demonstrating the beneficial sleep effect on MR learning, independently of the gender effect. Error bars indicate one SD.

In the re-test session, the number of correct items increased to 10.00 (±0.41) in the MNight group and 9.10 (±0.65) in the WNight group, while day groups made more errors (mean scores being 9.10±0.36 in the MDay and 8.00±0.62 in the WDay). An ANOVA_RM_ on the effect of the off-line consolidation yielded a session x consolidation interaction (F_1,36_ = 55.39, P<0.0001, *η_p_^2^* = 0.60), but no session x gender interaction (F_1,36_ = 0.24, P = 0.62, *η_p_^2^* = 0.006), no main effect of gender (F_1,36_ = 4.02, P = 0.05, *η_p_^2^* = 0.10), and no main effect of consolidation (F_1,36_ = 0.05, P = 0.81, *η_p_^2^* = 0.001). Bonferroni post-hoc comparisons revealed that the additional increase in performance between the post-training and re-test sessions in the Night groups was significant (P<0.0001), as was the decreased performance in the Day groups (P<0.001, Fig.3).

Response latencies were, in the re-test session, 12.55±0.87 s for the MNight group, 10.69±0.73 s in the MDay group, 11.70±0.82 s in the WNight group, and 13.11±1.27 s in the WDay group. The ANOVArm showed a main session effect (F_1,36_ = 22.06, P<0.0001, *η_p_^2^ = *0.38) and a gender x consolidation interaction (F_1,36_ = 6.05, P<0.05, *η_p_^2^* = 0.14), but no main effect of consolidation (F_1,36_ = 0.66, P = 0.42, *η_p_^2^* = 0.01) nor main effect of gender (F_1,36_ = 2.91, P = 0.09, *η_p_^2^* = 0.07). Post-hoc comparisons revealed only a difference between the MDay and the WDay groups at the post-training (P<0.05).

#### Questionnaire on MR Strategies

A convincing gender difference was only found for Questions 1, 2 and 3. Firstly, 85% of men reported that they rotated the entire figure while only 45% of women adopted the same strategy. Accordingly, 55% of women preferentially rotated only parts of the figure while only 10% of men used this strategy (*x^2^* = 12.22, *df* 1, P<0.05). In response to Question 2, a greater percentage of men (85%) than women (40%) reported using a non-verbal strategy (*x^2^* = 8.64, *df* 1, P<0.05). Finally, with regards to Question 3, 60% of women but no men stated that they used movements of the fingers or the hand to help with the rotation performance (*x^2^* = 17.25, *df* 1, P<0.05). For Question 4, 60% of the participants stated that they proceeded through all possible alternatives, and 50% (Question 5) that when they found a matching item, they compared the remaining options. Whereas when this questionnaire asked participants to report the processes and strategies used while performing MR, 72.5% answered that they tried various approaches to solve the problem (Question 6). Interestingly, 62% of the participants reported being more concerned with time limit when getting the right answers (Question 7). Finally, 87.2% declared that they were not totally sure with their answers before moving on to a next configuration (Question 8).

## Discussion

The present study was the first attempt to assess the offline consolidation effect after MR practice including a comparison between men and women. The main objective was twofold: (1) Does 3D VK-MR practice reduce the women *disadvantage*, and (2) Does the offline consolidation process during a night of sleep protract additional MR performance gains? First, we found an improvement in performance following the same amount of MR training in all participants. Interestingly, women tended to substantially enhance performance, although the gender difference remained significant. Second, we observed a delayed performance gains on MR after a night of sleep independently on gender, while an equivalent wake daytime period did not result in enhanced performance.

The common application of the VK-MR test [6] is a prototypical test for gender differences in spatial ability [4], [52], [53]. As expected, we confirmed such well-known differences since men outperformed women during the pre-training phase. Peters and Battista [54] argued that differences in experimental designs involving pairwise presentations, such as the VK test, might contribute to explain gender differences. Briefly, the authors underlined that time pressure, eye movements, as well as 2D vs. 3D items and the strategy adopted may differently impact gender MR performance. According to the MR questionnaire completed by participants in the present study, and in line with the existing literature [9], [51], [55], our data support that men and women employ different strategies to resolve MR problems, with women mentally rotating the items in an analytic piecemeal fashion and men prioritizing a more holistic strategy. Interestingly, data revealed greater performance in both men and women from the pre- to the post-training sessions, men outperforming women. Surprisingly, when directly comparing men and women performances during the post-training session, no significant gender difference was yielded, hence suggesting greater MR enhancement in women. This finding is in accordance with previous results showing the efficacy of MR training for women [‘‘post-training’’ session, 7,56,57,58,59,60]. In other words, as suggested by Kass, Ahlers, and Dugger [56], present findings tend to suggest that brief MR training may be sufficient to close the gap in MR performance between men and women. Jaušovec and Jaušovec [59] examined whether training can increase MR performance in women and change the related pattern of brain activity. They reported an increased activity in intraparietal regions, which was recently found to correlate to speed and accuracy of MR performance [61], [62], [63], though activation would remain stronger in men [13].

The most salient and novel finding from our study is that MR practice resulted in greater performance after a night of sleep, while a similar wake period rather induced a decrease in performance, regardless of gender. Specifically, participants subjected to a night of sleep significantly increased the number of correct responses and reduced response latencies. A reverse pattern of findings was observed in participants subjected to a similar wake daytime period. These findings strongly support the role of sleep in the offline (re)processing of memories [16], [64], as shown previously by experimental studies reporting the existence of delayed gains in performance after a night of sleep, but not after a comparable time interval during daytime [21], [22], [23], [24], [65], [66], [67], [68]. Interestingly, it has been suggested that when consolidation takes place, namely once the brain shifts from wake to sleep, it changes the organization of memories from being interactive to independent [36], hence challenging the concept of fixed independent memory systems. In other terms, explicit and implicit knowledge associated with a task interact during learning [69], and during the consolidation period they may interact or interfere over wake while they may evolve independently over sleep [35]. Here, participants used implicit process to perform the MR task [70], [71], as we did not ask them to engage MR but only to find the two correct responses, so that they dealt with explicit components provided both by the task instructions and the auto-elaborated strategies [72]. Thus, it seems plausible that explicit and implicit MR components were buffered and interacted in WM during MR practice, and then either interfered over wake or improved independently over the night of sleep. These findings are consistent with previous studies stating that when implicit skill and explicit declarative knowledge are acquired simultaneously, the subsequent offline motor processing depends on sleep [73]. In contrast, when an implicit skill is acquired with little or no declarative knowledge, the subsequent offline implicit skill processing occurs during wakefulness or sleep [74], [75]. Overall, these results describe, for the first time, that performance gains following MR are somewhat sleep dependent, regardless the gender of the participants.

It is worth noting that there was no significant difference in response latencies between pre- and post-training sessions, whereas they were respectively reduced after a night of sleep (re-test) and increased after wake in both men and women. Peters [52] underlined the question of the nature of differences in reaction time for a standard VK task, when presented on the computer screen in a way that is similar to that in the paper version. Here, for the first time, we assessed response latencies on a full five stimulus display, while we also included a time pressure (i.e. 30 s) to provide the answers. Such implementation of time constraint might have limited the potential effect provided by training on response latencies. Interestingly, whether early within-practice consolidation did not permit an enhancement in responses latencies, the night of sleep did, thus suggesting that the disturbing time constraint imposed in our task may have been challenged by the potent restructuration/facilitation process induced only by a night of sleep [36], [67]. Thus, as suggested by Peters [52], future work exploring the effect of training and consolidation in a VK task with a reaction time paradigm might provide new insights on gender differences.

Some limitations of this study must be mentioned. First, the night and daytime wake conditions did not exclude the possibility that lower performance after wake intervals resulted from variations of circadian rhythms and/or retroactive interference. Likewise, the possibility that the alteration of performance for Day groups could be partially related to fatigue cannot be excluded. Subjective data, however, tended to suggest that fatigue was not an issue, since a non-significant difference was reported in the SSS scores at the retest session. In addition, we asked participants to report their prevalent strategies to resolve the MR task but it is worth noting that 72.5% reported that they employed different approaches. Nevertheless, qualitative results were evident with respect to whether they rotated the whole figure (85% men) or only a specific section (65% women). Future research should now investigate how MR strategies evolve at different time points of the practice, i.e. at the beginning, after training and after consolidation.

To conclude, our findings support the causal role of sleep in improving MR performance in men and women, while a similar daytime period did not and even deteriorated performance. Although we cannot totally conclude on the beneficial effect of MR training to eliminate gender difference in performance, present data support that an adequate amount of MR training might allow women to reach an equivalent level of performance than men. Basically, both the nature and the level of complexity of the skill learning should be considered in greater details to determine the optimization of training protocols [68]. For example, Kuriyama, Stickgold, and Walker [67] demonstrated that greater delayed performance gains were observed following a night of sleep in motor skill procedures that were most difficult. Similar selective delayed gains were recently reported following explicit motor imagery of complex movements [76]. Based on these results, further investigations are necessary to determine whether the complexity of the MR task, i.e. the rate at which items can be rotated depends on the complexity of the stimulus, might in return be differently impacted by the sleep consolidation process. Future studies should also explore whether sleep can positively influence the performance of embodied spatial transformations, such as MR of abstract 3D shapes, including body characteristics [77], or even RM of body parts. Practically, these findings might have fruitful applications in rehabilitation and motor learning processes, in which MR training of body parts is frequently employed in order to assess a deficit in cognitive representation impairment [41], [78], [79], [80], or to improve motor performance [81], [82].

## Supporting Information

Table S1 **Number of correct responses and response latencies.**
(DOC)

Appendix S1 **Questionnaire used to identify the MR strategies.**
(DOC)

## References

[1] Shepard RN, Metzler J (1971) Mental rotation of three-dimensional objects. Science 171: 701–703. 5540314 10.1126/science.171.3972.701

[2] Carballis MC, McLaren R (1982) Interaction between perceived and imagined rotation. J Exp Psychol Hum Percept Perform 8: 215–224. 6461718 10.1037//0096-1523.8.2.215

[3] Shepard RN, Metzler J (1982) Mental images and their transformation. Cambridge, MA: MIT Press/Bradford Books.

[4] Voyer D, Voyer S, Bryden MP (1995) Magnitude of sex differences in spatial abilities: a meta-analysis and consideration of critical variables. Psychol Bull 117: 250–270. 7724690 10.1037/0033-2909.117.2.250

[5] Guillot A, Champely S, Batier C, Thiriet P, Collet C (2007) Relationship between spatial abilities, mental rotation and functional anatomy learning. Adv Health Sci Educ Theory Pract 12: 491–507. 16847728 10.1007/s10459-006-9021-7

[6] Vandenberg SG, Kuse AR (1978) Mental rotations, a group test of three-dimensional spatial visualization. Percept Mot Skills 47: 599–604. 724398 10.2466/pms.1978.47.2.599

[7] Neubauer AC, Bergner S, Schatz M (2010) Two- vs. three-dimensional presentation of mental rotation tasks: Sex differences and effects of training on performance and brain activation. Intelligence 38: 529–539. 20953415 10.1016/j.intell.2010.06.001 PMC2940390

[8] Jordan K, Wustenberg T, Heinze HJ, Peters M, Jancke L (2002) Women and men exhibit different cortical activation patterns during mental rotation tasks. Neuropsychologia 40: 2397–2408. 12417468 10.1016/s0028-3932(02)00076-3

[9] Heil M, Jansen-Osmann P (2008) Sex differences in mental rotation with polygons of different complexity: Do men utilize holistic processes whereas women prefer piecemeal ones? Q J Exp Psychol (Hove) 61: 683–689. 18421643 10.1080/17470210701822967

[10] Yu Q, Tang Y, Li J, Lu Q, Wang H, et al (2009) Sex differences of event-related potential effects during three-dimensional mental rotation. Neuroreport 20: 43–47. 19057281 10.1097/WNR.0b013e32831c50f4

[11] Butler T, Imperato-McGinley J, Pan H, Voyer D, Cordero J, et al (2006) Sex differences in mental rotation: top-down versus bottom-up processing. Neuroimage 32: 445–456. 16714123 10.1016/j.neuroimage.2006.03.030

[12] Clements-Stephens AM, Rimrodt SL, Cutting LE (2009) Developmental sex differences in basic visuospatial processing: differences in strategy use? Neurosci Lett 449: 155–160. 19000742 10.1016/j.neulet.2008.10.094 PMC2627765

[13] Semrud-Clikeman M, Fine JG, Bledsoe J, Zhu DC (2012) Gender Differences in Brain Activation on a Mental Rotation Task. Int J Neurosci. 10.3109/00207454.2012.693999 22651549

[14] Zacks JM (2008) Neuroimaging studies of mental rotation: a meta-analysis and review. J Cogn Neurosci 20: 1–19. 17919082 10.1162/jocn.2008.20013

[15] Eisenegger C, Herwig U, Jancke L (2007) The involvement of primary motor cortex in mental rotation revealed by transcranial magnetic stimulation. Eur J Neurosci 25: 1240–1244. 17331219 10.1111/j.1460-9568.2007.05354.x

[16] Stickgold R, Walker MP (2007) Sleep-dependent memory consolidation and reconsolidation. Sleep Med 8: 331–343. 17470412 10.1016/j.sleep.2007.03.011 PMC2680680

[17] Karni A, Tanne D, Rubenstein BS, Askenasy JJ, Sagi D (1994) Dependence on REM sleep of overnight improvement of a perceptual skill. Science 265: 679–682. 8036518 10.1126/science.8036518

[18] Stickgold R, James L, Hobson JA (2000) Visual discrimination learning requires sleep after training. Nat Neurosci 3: 1237–1238. 11100141 10.1038/81756

[19] Gaab N, Paetzold M, Becker M, Walker MP, Schlaug G (2004) The influence of sleep on auditory learning: a behavioral study. Neuroreport 15: 731–734. 15094486 10.1097/00001756-200403220-00032

[20] Fischer S, Hallschmid M, Elsner AL, Born J (2002) Sleep forms memory for finger skills. Proc Natl Acad Sci U S A 99: 11987–11991. 12193650 10.1073/pnas.182178199 PMC129381

[21] Walker MP, Brakefield T, Morgan A, Hobson JA, Stickgold R (2002) Practice with sleep makes perfect: sleep-dependent motor skill learning. Neuron 35: 205–211. 12123620 10.1016/s0896-6273(02)00746-8

[22] Walker MP, Brakefield T, Seidman J, Morgan A, Hobson JA, et al (2003) Sleep and the time course of motor skill learning. Learn Mem 10: 275–284. 12888546 10.1101/lm.58503 PMC202318

[23] Debarnot U, Castellani E, Valenza G, Sebastiani L, Guillot A (2011) Daytime naps improve motor imagery learning. Cogn Affect Behav Neurosci 11: 541–50. 21842279 10.3758/s13415-011-0052-z

[24] Debarnot U, Creveaux T, Collet C, Doyon J, Guillot A (2009) Sleep contribution to motor memory consolidation: a motor imagery study. Sleep 32: 1559–1565. 20041591 10.1093/sleep/32.12.1559 PMC2786039

[25] Debarnot U, Maley L, Rossi DD, Guillot A (2010) Motor interference does not impair the memory consolidation of imagined movements. Brain Cogn 74: 52–57. 20621408 10.1016/j.bandc.2010.06.004

[26] Maquet P, Laureys S, Peigneux P, Fuchs S, Petiau C, et al (2000) Experience-dependent changes in cerebral activation during human REM sleep. Nat Neurosci 3: 831–836. 10903578 10.1038/77744

[27] Nishida M, Walker MP (2007) Daytime naps, motor memory consolidation and regionally specific sleep spindles. PLoS One 2: e341. 17406665 10.1371/journal.pone.0000341 PMC1828623

[28] Buhry L, Azizi AH, Cheng S (2011) Reactivation, replay, and preplay: how it might all fit together. Neural Plast 2011: 203462. 21918724 10.1155/2011/203462 PMC3171894

[29] Walker MP, Stickgold R (2004) Sleep-dependent learning and memory consolidation. Neuron 44: 121–133. 15450165 10.1016/j.neuron.2004.08.031

[30] Walker MP, Stickgold R (2010) Overnight alchemy: sleep-dependent memory evolution. Nat Rev Neurosci 11: 218; author reply 218. 10.1038/nrn2762-c1 PMC2891532 20168316

[31] Kuriyama K, Mishima K, Suzuki H, Aritake S, Uchiyama M (2008) Sleep accelerates the improvement in working memory performance. J Neurosci 28: 10145–10150. 18829972 10.1523/JNEUROSCI.2039-08.2008 PMC6671268

[32] Colom R, Jung RE, Haier RJ (2007) General intelligence and memory span: evidence for a common neuroanatomic framework. Cogn Neuropsychol 24: 867–878. 18161499 10.1080/02643290701781557

[33] Walker MP (2009) The role of sleep in cognition and emotion. Ann N Y Acad Sci 1156: 168–197. 19338508 10.1111/j.1749-6632.2009.04416.x

[34] Genzel L, Kiefer T, Renner L, Wehrle R, Kluge M, et al (2012) Sex and modulatory menstrual cycle effects on sleep related memory consolidation. Psychoneuroendocrinology 37: 987–998. 22153362 10.1016/j.psyneuen.2011.11.006

[35] Brown RM, Robertson EM (2007) Off-line processing: reciprocal interactions between declarative and procedural memories. J Neurosci 27: 10468–10475. 17898218 10.1523/JNEUROSCI.2799-07.2007 PMC6673170

[36] Robertson EM (2012) New insights in human memory interference and consolidation. Curr Biol 22: R66–71. 22280913 10.1016/j.cub.2011.11.051 PMC3267959

[37] Wang B, Fu XL (2009) Gender difference in the effect of daytime sleep on declarative memory for pictures. J Zhejiang Univ Sci B 10: 536–546. 19585672 10.1631/jzus.B0820384 PMC2704972

[38] Marusan M, Kulistak J, Zara J (2006) Virtual Reality in neurorehabilitation: mental rotation. In: Press VPU, editor. Proceedings of the Third Central European Multimedia and Virtual Reality Conference. pp. 77-83.

[39] Rose FF, Atree EA, Brooks BM, Johnson DA (1998) Virtual environments in brain damage rehabilitation: a rationale from basic neuroscience. In: Giuseppe Riva BKW, Enrico Molinari., editor. Virtual environments in clinical Psychology and Neuroscience. Amsterdam, Netherlands. 10350924

[40] Tomasino B, Rumiati RI (2004) Effects of strategies on mental rotation and hemispheric lateralization: neuropsychological evidence. J Cogn Neurosci 16: 878–888. 15200714 10.1162/089892904970753

[41] Sharma N, Pomeroy VM, Baron JC (2006) Motor imagery: a backdoor to the motor system after stroke? Stroke 37: 1941–1952. 16741183 10.1161/01.STR.0000226902.43357.fc

[42] Zacks J, Rypma B, Gabrieli JD, Tversky B, Glover GH (1999) Imagined transformations of bodies: an fMRI investigation. Neuropsychologia 37: 1029–1040. 10468366 10.1016/s0028-3932(99)00012-3

[43] Podzebenko K, Egan GF, Watson JD (2005) Real and imaginary rotary motion processing: functional parcellation of the human parietal lobe revealed by fMRI. J Cogn Neurosci 17: 24–36. 15701237 10.1162/0898929052879996

[44] Wexler M, Kosslyn SM, Berthoz A (1998) Motor processes in mental rotation. Cognition 68: 77–94. 9775517 10.1016/s0010-0277(98)00032-8

[45] Oldfield RC (1971) The assessment and analysis of handedness: the Edinburgh inventory. Neuropsychologia 9: 97–113. 5146491 10.1016/0028-3932(71)90067-4

[46] Buysse DJ, Reynolds CF, 3rd, Monk TH, Berman SR, Kupfer DJ (1989) The Pittsburgh Sleep Quality Index: a new instrument for psychiatric practice and research. Psychiatry Res 28: 193–213. 2748771 10.1016/0165-1781(89)90047-4

[47] Agniel A, Joanette Y, Doyon B, Duchein C (1987) Protocole d’évaluation des gnosies visuelles Montréal-Toulouse. In: Laboratoire Th.-Alajouanine CdrdCHC-d-N, editor.

[48] Warrington EK, James M (1991) The visual object and space perception battery.; Company TVT, editor. Bury St. Edmunds UK.

[49] Hoddes E, Dement WC, Zarcone V (1972) The development and use of the Stanford sleepiness scale. Psychophysiology 9: 150.

[50] Linn MC, Peterson AC (1986) A meta-analysis of gender differences in spatial ability: Implications for mathematics and science achievement. In: Linn JSHMC, editor. The Psychology of Gender: Advances through meta-analysis Baltimore: The Johns Hopkins University Press. pp. 67-101.

[51] Peters M, Laeng B, Latham K, Jackson M, Zaiyouna R, et al (1995) A redrawn Vandenberg and Kuse mental rotations test: different versions and factors that affect performance. Brain Cogn 28: 39–58. 7546667 10.1006/brcg.1995.1032

[52] Peters M (2005) Sex differences and the factor of time in solving Vandenberg and Kuse mental rotation problems. Brain Cogn 57: 176–184. 15708213 10.1016/j.bandc.2004.08.052

[53] Peters M, Manning JT, Reimers S (2007) The effects of sex, sexual orientation, and digit ratio (2D:4D) on mental rotation performance. Arch Sex Behav 36: 251–260. 17394056 10.1007/s10508-006-9166-8

[54] Peters M, Battista C (2008) Applications of mental rotation figures of the Shepard and Metzler type and description of a mental rotation stimulus library. Brain Cogn 66: 260–264. 17967499 10.1016/j.bandc.2007.09.003

[55] Parsons TD, Larson P, Kratz K, Thiebaux M, Bluestein B, et al (2004) Sex differences in mental rotation and spatial rotation in a virtual environment. Neuropsychologia 42: 555–562. 14728927 10.1016/j.neuropsychologia.2003.08.014

[56] Kass SJ, Ahlers RH, Dugger M (1998) Eliminating gender differences through practice in an applied visual spatial task. Human Performance 11: 337–349.

[57] Sanz de Acedo Lizarraga ML, García Ganuza JM (2003) Improvement of mental rotation in girls and boys. Sex Roles 49: 277–286.

[58] Koscik T, O'Leary D, Moser DJ, Andreasen NC, Nopoulos P (2009) Sex differences in parietal lobe morphology: relationship to mental rotation performance. Brain Cogn 69: 451–459. 18980790 10.1016/j.bandc.2008.09.004 PMC2680714

[59] Jaušovec N, Jaušovec K (2012) Sex differences in mental rotation and cortical activation patterns: Can training change them? Intelligence 40: 151–162.

[60] Feng J, Spence I, Pratt J (2007) Playing an action video game reduces gender differences in spatial cognition. Psychol Sci 18: 850–855. 17894600 10.1111/j.1467-9280.2007.01990.x

[61] Semrud-Clikeman M, Goldenring Fine J, Bledsoe J, Zhu DC (2012) Gender Differences in Brain Activation on a Mental Rotation Task. International Journal of Neuroscience doi:10.3109/00207454.2012.693999. 10.3109/00207454.2012.693999 22651549

[62] Hoppe C, Fliessbach K, Stausberg S, Stojanovic J, Trautner P, et al (2012) A key role for experimental task performance: effects of math talent, gender and performance on the neural correlates of mental rotation. Brain Cogn 78: 14–27. 22088776 10.1016/j.bandc.2011.10.008

[63] Jordan K, Wustenberg T (2010) The neural network of spatial cognition and its modulation by biological and environmental factors. Journal of Individual Differences 3: 83–90.

[64] Maquet P (2004) A role for sleep in the processing of memory traces. Contribution of functional neuroimaging in humans. Bull Mem Acad R Med Belg 159: 167–170. 15615089

[65] Fischer S, Nitschke MF, Melchert UH, Erdmann C, Born J (2005) Motor memory consolidation in sleep shapes more effective neuronal representations. J Neurosci 25: 11248–11255. 16339020 10.1523/JNEUROSCI.1743-05.2005 PMC6725908

[66] Karni A (1995) When practice makes perfect. Lancet 345: 395. 10.1016/s0140-6736(95)90386-0 7845149

[67] Kuriyama K, Stickgold R, Walker MP (2004) Sleep-dependent learning and motor-skill complexity. Learn Mem 11: 705–713. 15576888 10.1101/lm.76304 PMC534699

[68] Doyon J, Korman M, Morin A, Dostie V, Hadj Tahar A, et al (2009) Contribution of night and day sleep vs. simple passage of time to the consolidation of motor sequence and visuomotor adaptation learning. Exp Brain Res 195: 15–26. 19277618 10.1007/s00221-009-1748-y PMC2752878

[69] Willingham DT, Goedert KM (1999) The relation between implicit and explicit learning: Evidence for parallel development. Psychol Sci 10: 531–534.

[70] de Lange FP, Roelofs K, Toni I (2008) Motor imagery: a window into the mechanisms and alterations of the motor system. Cortex 44: 494–506. 18387583 10.1016/j.cortex.2007.09.002

[71] Prime DJ, Jolicoeur P (2010) Mental rotation requires visual short-term memory: evidence from human electric cortical activity. J Cogn Neurosci 22: 2437–2446. 19702462 10.1162/jocn.2009.21337

[72] Berns GS, Cohen JD, Mintun MA (1997) Brain regions responsive to novelty in the absence of awareness. Science 276: 1272–1275. 9157889 10.1126/science.276.5316.1272

[73] Robertson EM (2009) From creation to consolidation: a novel framework for memory processing. PLoS Biol 7: e19. 19175290 10.1371/journal.pbio.1000019 PMC2631067

[74] Spencer RM, Sunm M, Ivry RB (2006) Sleep-dependent consolidation of contextual learning. Curr Biol 16: 1001–1005. 16713957 10.1016/j.cub.2006.03.094

[75] Robertson EM, Pascual-Leone A, Press DZ (2004) Awareness modifies the skill-learning benefits of sleep. Curr Biol 14: 208–212. 14761652 10.1016/j.cub.2004.01.027

[76] Debarnot U, Castellani E, Guillot A (In press) Selective delayed gains following motor imagery of complex movements. Archives Italiennes de biologie. 10.4449/aib.v150i4.1394 23479457

[77] Amorim MA, Isableu B, Jarraya M (2006) Embodied spatial transformations: "body analogy" for the mental rotation of objects. J Exp Psychol Gen 135: 327–347. 16846268 10.1037/0096-3445.135.3.327

[78] Curtze C, Otten B, Postema K (2010) Effects of lower limb amputation on the mental rotation of feet. Exp Brain Res 201: 527–534. 19902193 10.1007/s00221-009-2067-z PMC2832871

[79] Fiorio M, Tinazzi M, Ionta S, Fiaschi A, Moretto G, et al (2007) Mental rotation of body parts and non-corporeal objects in patients with idiopathic cervical dystonia. Neuropsychologia 45: 2346–2354. 17412373 10.1016/j.neuropsychologia.2007.02.005

[80] Harris IM, Harris JA, Caine D (2002) Mental-rotation deficits following damage to the right basal ganglia. Neuropsychology 16: 524–537. 12382991 10.1037//0894-4105.16.4.524

[81] Stransky D, Wilcox LM, Dubrowski A (2010) Mental rotation: cross-task training and generalization. J Exp Psychol Appl 16: 349–360. 21198252 10.1037/a0021702

[82] Hoyek N, Collet C, Rastello O, Fargier P, Thiriet P, et al (2009) Enhancement of mental rotation abilities and its effect on anatomy learning. Teach Learn Med 21: 201–206. 20183339 10.1080/10401330903014178

