# Supplementary material for: Mental Rotation: Effects of Gender, Training and Sleep Consolidation
Source: PLoS One. 2013 Mar 27;8(3):e60296. doi: 10.1371/journal.pone.0060296 (PMC3609807; doi:10.1371/journal.pone.0060296)
Supplement: Table S1 — Number of correct responses and response latencies. (DOC) [file pone.0060296.s001.doc]

Table S1

|  | Accuracy /12 | | | Time of the correct answers (s) | | |  |
| --- | --- | --- | --- | --- | --- | --- | --- |
|  | Pre-training | Post-training | Re-test | Pre-training | Post-training | Re-test |  |
| MNight | 6.20 (.78) | 8.80 (.34) | 10.00 (.41) | 15.85 (1.24) | 14.96 (1.22) | 12.55 (.87) |  |
| MDay | 8.10 (.57) | 10.10 (.53) | 9.10 (.36) | 11.83 (.95) | 11.20 (.81) | 10.69 (.73) |  |
| WNight | 5.20 (.92) | 7.70 (.81) | 9.10 (.65) | 15.97 (1.75) | 14.36 (.86) | 11.70 (.82) |  |
| WDay | 5.40 (.47) | 8.90 (.74) | 8.00 (.62) | 14.92 (1.48) | 15.67 (1.13) | 13.11 (1.27) |  |
