# Supplementary material for: Mental Rotation: Effects of Gender, Training and Sleep Consolidation
Source: PLoS One. 2013 Mar 27;8(3):e60296. doi: 10.1371/journal.pone.0060296 (PMC3609807; doi:10.1371/journal.pone.0060296)
Supplement: Appendix S1 — Questionnaire used to identify the MR strategies. (DOC) [file pone.0060296.s002.doc]

Appendix S1

**1)**

- I rotated the whole figure in my mind when making the comparison
- I rotated only a specific section of the figure in my mind when making the comparison
- I am not sure how exactly I did it
- Other (explain)

**2)**

- I thought through the steps verbally in my mind (i.e. “two cubes up and three down”)
- I mainly relied on visualizing the figures and did not talk myself through the steps
- I am not sure

**3)**

- I used movements of my finger, hand and/or pencil to help me with the task
- I did not use any movement of my finger, hand and/or pencil to help me with the task

**4)**

- I scanned all possible alternative before answering and then made my choice
- I systematically went through all possible alternatives, trying the first, then the second etc.
- I went through the possible alternatives in a haphazard and nonsystematic way
- Other (specify)

**5)**

- I always compared all test items to the target figure
- Once I found a matching item, I compared the rest of options to the match
- I did a bit of both

**6)**

- I developed a specific approach to solve the problems
- I tried various approaches to solve the problems
- I had no specific approach

**7)**

- I was more concerned with the time limit than I was about getting the right answers
- I was more concerned with getting the right answers than I was about time limit
- I was more concerned with getting all the answers completed than I was about getting the correct answers
- I did not care how I did

**8)**

- I double checked my answers before moving on to the next problem
- I was vaguely confident of my answers before I moved to the next problem
- I was unsure of my answers before moving on to the next problem
- I guessed most of the time
